# A Mobile App to Support Clinical Diagnosis of Upper Respiratory Problems (eHealthResp): Co-Design Approach

**DOI:** 10.2196/19194

**Published:** 2021-01-28

**Authors:** João Moura, Ana Margarida Pisco Almeida, Fátima Roque, Adolfo Figueiras, Maria Teresa Herdeiro

**Affiliations:** 1 Department of Medical Sciences iBiMED—Institute of Biomedicine University of Aveiro Aveiro Portugal; 2 Department of Communication and Art/DigiMedia University of Aveiro Aveiro Portugal; 3 Research Unit for Inland Development Polytechnic Institute of Guarda (UDI/IPG) Guarda Portugal; 4 Health Sciences Research Centre University of Beira Interior (CICS-UBI) Covilhã Portugal; 5 Department of Preventive Medicine and Public Health University of Santiago de Compostela Santiago de Compostela Spain; 6 Consortium for Biomedical Research in Epidemiology and Public Health (CIBER Epidemiology and Public Health-CIBERESP) Madrid Spain; 7 Health Research Institute of Santiago de Compostela (IDIS) Santiago de Compostela Spain

**Keywords:** mHealth, Clinical Decision Support Systems, respiratory system, diagnose, interface, mobile phone

## Abstract

**Background:**

The misuse of antibiotics is a global public health issue that fosters bacterial resistance and jeopardizes generational health. The development of validated tools such as web-based courses and mobile apps to enhance clinical decisions in upper respiratory infections is of great importance in reducing the incorrect use of antibiotics in these situations.

**Objective:**

The aim of this study was to design and prevalidate the interface of a mobile app to assist and provide clinical support in the diagnosis of upper respiratory problems. We aimed to assess the adequacy and usability of the interface of the tool in the belief that it could be beneficial to health care delivery in the clinical decision setting.

**Methods:**

Using a co-design approach that brought together professionals in interface design and experts in pharmacology and pharmacoepidemiology, the mobile app interface was evaluated through peer review sessions held by interface design professionals on a heuristic survey. The reviewers accessed a high-fidelity interactive mock-up of the interface and filled in a questionnaire to assess the dimensions of *layout and visual design* and *navigation and tasks*. The resulting feedback of this evaluation supported the redesign of the primary interface, which was assessed for the second time by 2 of the previously mentioned reviewers.

**Results:**

With 4 as the highest score, the interface scored a mean of 3.16 (SD 0.45; median of the means 3.2) for *layout and visual design* and a mean of 3.43 (SD 0.33; median of the means 3.51) for *navigation and tasks,* reflecting an overall positive evaluation. The open-ended commentaries allowed us to better understand specific recommendations of the reviewers. Throughout this section, approximately 0.98 comments per parameter were registered, reflecting a high level of effectiveness of the chosen parameters in identifying potential problems. The resultant beta version of the interface, addressing the majority of the detected problems, was further assessed by 2 of the previous reviewers, validating the new design. Future tests with physicians and pharmacists will help assess credibility and user experience dimensions.

**Conclusions:**

Our study revealed that the designed interface is easy to interpret and use. Peer reviewers raised important issues that could be easily fixed and positively reassessed. As a result, the study enabled us to produce a new tool for interface usability assessment and a set of recommendations for developing mobile interfaces for clinical decision support systems in the scope of upper respiratory problems.

## Introduction

### Background

Antibiotic resistance is a major public health problem worldwide that is mostly fostered by inappropriate use of antibiotic medications. At the same time, data and advances in health care are growing not only in quantity but also in complexity; thus, health systems, practitioners, and even patients are required to be in a constant learning state to achieve effective monitoring and evaluation [1].

With the near total ubiquity of mobile technologies, mobile health (mHealth) is becoming an increasingly established field with important results in different domains [2-4]. This gives rise to new possibilities in enhancing clinical decisions in all medical fields, including antibiotic prescription and dispensing.

In this framework, the project eHealthResp proposes to create and evaluate eHealth tools to support clinical decisions and patient empowerment in the management of upper respiratory infections. These tools include an web-based course targeted toward physicians and pharmacists and a clinical decision support system (CDSS) mobile app targeted toward physicians, pharmacists, and patients.

At a later stage of the research, both tools will be used by participants of an educational intervention about antibiotic prescription and dispensing for upper respiratory symptoms supported by the eHealthResp project.

Expected outcomes include a decrease and improvement in the use of antibiotics as well as a comprehensive list of guidelines in designing and implementing feasible and usable tools for CDSS in a broad scope, particularly for cases involving the upper respiratory system.

In the realm of these predictions, the work in progress and the main issue of this paper focus on the methodology and subsequent results within the interface design stage of the mobile app tool for smartphones running Android or iOS operative systems.

**Figure 1:**
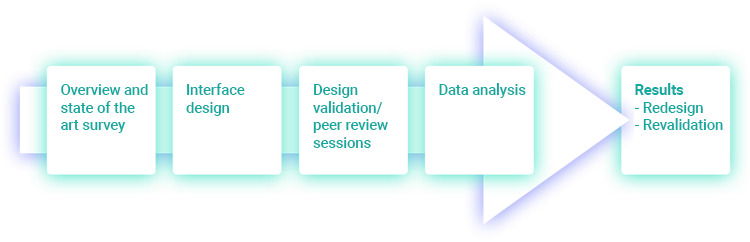
Design of the research.

This research stage can be summarized within the following 4 components:

- Goals:
  The goals of the research are to develop the interface of a CDSS tool that supports decisions while prescribing and dispensing antibiotics in cases involving upper respiratory system symptoms, evaluate its usability, and measure how well the human-computer interaction (HCI) experts in the peer review sessions perceive the interface in terms of effective, efficient, and satisfactory use [5].
- Conceptual framework
  The incorrect use of antibiotics represents the main worldwide factor for the increasing bacterial resistance to these drugs, requiring a more efficient approach to prescription and dispensing processes.
  There is a need to use an interdisciplinary approach and co-design methodologies when developing mobile health interfaces.
- Research question
  How can we develop an interface for an app to support physicians, pharmacists, and patients to properly use previously validated algorithms for upper respiratory symptoms?
- Methods
  The development process was organized in 4 steps (Figure 1): (1) a primary step regarding a literature search and state-of-the-art analysis, followed by (2) the design of the interface by a team of experts, namely 2 HCI practitioners and 3 experts in pharmacology and pharmacoepidemiology, resulting in the alpha version of the interface. This version went through a prevalidation of the developed interface throughout (3) peer review sessions in a heuristic style evaluation [6] executed by 5 HCI practitioners with experience in interface design and availability. Following the tests, (4) the data analysis stage took into account the experts’ outcome and produced the interface redesign (beta version), including a new validation phase, leading to the final proposal.

### eHealthResp Mobile App

Currently targeted toward Android and iOS mobile devices, the eHealthResp mobile app is under development and consists of a diagnostic tool designed to enhance the clinical decision set facing upper respiratory symptoms. The algorithm behind the app and a version of the app were previously validated and designed for the pharmaceutical scope by a research group from the University of Santiago de Compostela based on the work of Molero et al [7]. This served as the basis for the development of the new proposal.

At the end-user level, the tool consists of a wizard that guides the user through the dichotomous key-like algorithm and, in cases in which a disease is identified, presents the end results in a diagnosis format, including *know more*, *treatment*, *prognosis* and *when to derive* information. The workflow was kept linear, straightforward, and with little to no deviations from the main course to keep the user’s focus on the diagnosis.

Although the previously developed app, available on the Google Play store, presents the wizard at a functional level, the interface design challenge was not considered when it was developed. At the design level, the major weaknesses of the app concern the use of stretched pictograms, misuse of Android navigation elements (eg, tabs, buttons), lack of consistency between pages and graphical elements (eg, buttons, pictograms, backgrounds), unoptimized image compression, and an overall assessment reflecting an unpleasant interface that fails to entice the user to trust and reuse the tool [8,9].

## Methods

### Overview and State of the Art

Using the Scopus database, a combination of the keywords *mobile app* (or *mobile health* or *app*), *clinical decision*, and *respiratory* was used to search for similar studies.

Due to the lack of direct references concerning diagnosis apps and design for mHealth, complementary searches were held combining the terms *algorithm*, *mobile*, *design*, *diagnosis*, or *diagnose*, and *respiratory*. From this search, 9 other articles were selected based on title and keywords. In total, 47 articles were selected and analyzed.

In addition to this review, a nonexhaustive benchmark-like search was conducted to gather a glimpse of the state of art in interface and design options for apps in the respiratory and CDSS fields. For this, separate searches were held on the tags *respiratory* and *clinical decision* in the 2 most relevant app stores (Google Play and App Store). For both the tags and stores, a subsearch for paid apps was performed.

The inclusion criteria were as follows: (1) respiratory system representations, (2) clinical decision tools, (3) quiz formats, and (4) informational or educational content involving the respiratory system. Apps were listed if they included at least one of the previous criteria. Apps that had cross-functionalities with the app under development were highlighted.

For each app, the list included inherent details (title, icon, link, creator, classification, price, description, last update, size, number of downloads, and screenshots) and 2 evaluation lists (pro- and counter-considerations) of the interface characteristics concerning the given screenshots and listed functionalities and based on the literature review. When available for free, highlighted apps were downloaded for a deeper analysis.

### Interface Design

**Figure 2:**
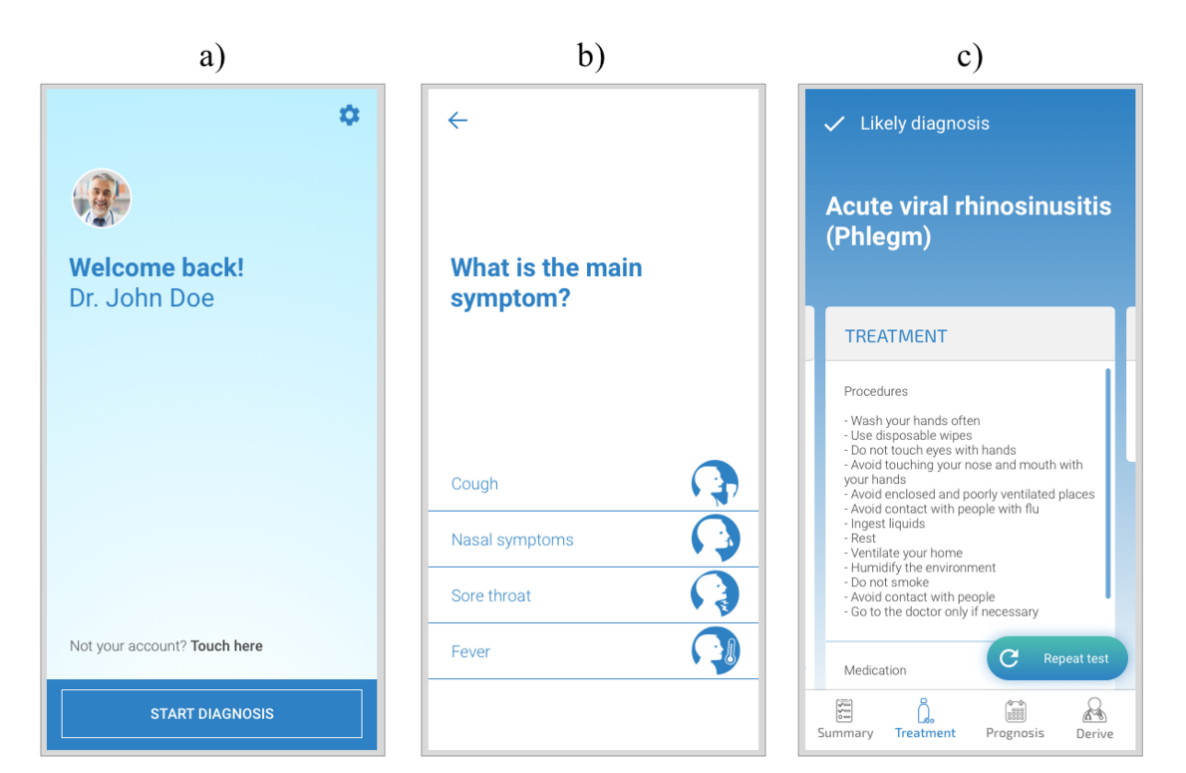
Light theme interface design examples before validation. (a) Homepage; (b) first question page; (c) likely diagnosis page.

To achieve an end design that suits the app users (physicians and pharmacists), the interdisciplinary team involved in this project worked together toward the development of the alpha version of the interface (Figure 2). In addition to periodic meetings, a questionnaire was used to assess the opinions of experts in pharmacology and pharmacoepidemiology about different interface solutions, assigning experience-based values throughout a participatory strategy [10]. This includes understanding the needs and preferences of experts and their peers (physicians and pharmacists) regarding content (script and imagery) and visualization formats. Using fast prototyping tools (Sketch and Marvel App), high-fidelity interface mock-ups were made according to participants’ previous feedback and presented to the participants.

To limit the time spent in developing high-fidelity mock-ups, the design covered strictly necessary paths to walkthrough the most decisive type-pages. Redundant, secondary, and broadly studied representations were avoided, such as pages with the same structure but different content and the Settings page.

### Peer Review Sessions

To attain an outsider point-of-view and opinion, a first series of peer review sessions was held by a group of 5 experts in interface design. This group was conveniently gathered based on the availability and diversity of the members’ academic and professional backgrounds.

After an introduction to the research framework and approach, each expert reviewer received a *PDF* document with the interface mock-up pages and an interactive high-fidelity mock-up version of the interface in the web-based app Marvel App. Both the *PDF* document and the Marvel mock-up presented 2 walkthrough paths: one for a light version of the interface, that is, brighter, and the other in a darker mode. To assess the quality of the navigation and graphical design of the interface, a ready-to-fill spreadsheet was provided. This document, an adapted version of the web-assessment tools, *38 page layout and visual design usability guidelines* and *29 navigation and IA usability guidelines* (IA stands for *information architecture*), both part of the “247 web usability guidelines” by David Travis [11]. The guidelines provided by Travis for web usability consist of bullet point lists that are meant to be used as assessment tools to improve consistency and design practices. As the author emphasizes, these guidelines could and should be adapted to the specific context in scope. The assessment tool was translated from English to Portuguese and adapted to keep the focus on the mobile app scope and the 2 main dimensions concerning interface and usability: (1) layout and visual design and (2) navigation and tasks. The adapted version presents a set of 30 parameters to assess layout and visual design and 21 parameters for navigation and tasks. Each parameter presented a statement suggesting a good assessment for a specific criterion (eg, “1. Content density is appropriate for target users and relevant tasks”). The reviewer had the possibility to classify each parameter through an adapted version of the Likert scale, measuring from 1 (Do not agree) to 4 (Totally agree), so that the design team could perceive the importance of the parameters in a more hierarchical way and determine whether to ignore or pay attention to each parameter. The choice for the lack of a fifth neutral middle value was taken to force the reviewer’s positioning, as applied in the original binary (complies or does not comply) guideline tool by Travis. Similar to the original tool, each parameter had an open-ended space for observations.

### Data Analysis

The mean and median were calculated to interpret the quantitative data collected throughout the reviewers’ evaluation using the adapted Likert scale for each bullet point. For the qualitative information gathered through the commentaries, a mean median was calculated to express the number of commentaries by bullet point. Given the relatively small sample of reviewers, every commentary was taken into consideration regarding the specific layout elements that were being referred to and the importance of the issue being addressed.

## Results

### Overview and State of the Art

The search was conducted between February and April 2019, and a total of 297 references were found. Only 34 articles directly addressed or focused on the searched issues. These 34 articles comprised a publishing period between 2004 and 2019. In total, 9 of the articles found describe studies conducted by the same organization—Allergic Rhinitis and its Impact on Asthma (ARIA) [12-20].

Some of the studies directly addressed the research framework (mobile interface design for CDSS in respiratory conditions); the majority of these focused on the effectiveness of a particular app in enhancing diagnosis and prescription, but few of them consider the user experience with the interface and the hedonic qualities of the interface that influence its long-term use [21]. Nonetheless, they gave important hints for the production of valuable and understandable tools.

The following list presents the main results of the overview, with the articles found and 13 other references that were derived from the original 34:

1. Evidence of mobile phones as potentially useful tools for health purposes [12,14,17,20,22-42].
2. List of state-of-the-art apps in the health field [43-45] and app ratings services [28], further used in the state-of-the-art review.
3. Guidelines for implementing CDSS using smartphones [13,14,17-19,27,30,36,44,46-52].
4. Guidelines for Android design [53] and HCI design [11,46,48,54-59].

The state-of-the-art search covered a period between March 20 and 30, 2019, and produced a list of 98 apps (34 on the App store and 64 on the Play Store. In total, 15 of these apps, were highlighted and 1 was found in both stores).

Overall, the analyzed apps exhibited the following identified characteristics:

- A chat allegory or personal assistant.
- Credibility through minimalism.
- Good visibility of the system.
- Use of validated standards and algorithms.
- Recognizable imagery.
- Use of the system norms.
- Paid apps did not reflect better design or more validated resources.

### Interface Design

As already mentioned, this stage produced a new interface proposal featuring one static and one interactive mock-up. This design took into account the guidelines found during the literature search and state-of-the-art survey stage, the CDSS tool requirements expressed through the previously developed app (eRes), and the researchers’ considerations. Among the considerations, the practitioners suggested the design of 2 themed versions (*light* and *dark*), for the purposes of personalization and comparison of the visibility of the layout structure. Each version consists mostly of a color switch between the content page and the background color. The secondary or action color was maintained, and the problem or error color was adjusted to improve its visibility.

Further considering the color issue, different chromatic choices were used to visually distinguish the interfaces for different users (physicians, pharmacists, and patients).

To convey the project aesthetic and credibility standards, the original interface layout was broadly redesigned, including reconsideration of the interface elements and their positioning. This includes not only the color aspect but also the typeface, buttons, animations, and redesign of the illustrative pictograms. Small tweaks to the navigation structure were performed on the Results page to gather related information, highlight important content, and hide secondary information in secondary pages.

### Peer Review Sessions

The input given was used to produce the beta version and included the reconsideration of gradients (to be avoided), layout (repositioning elements such as questions and answers and buttons such as *Repeat test*), pictograms (redesigned and avoided in some cases), typefaces (avoiding bold and light versions), and the design of a confirmation page before finishing the test.

After the redesign, the new interface was presented in person through meetings with 2 of the reviewers, who were selected based on the amount of input given previously. These meetings were also intended to ensure that the design team correctly interpreted their responses and assessed whether the new proposals effectively rectified the issues that were found.

### Data Analysis

With 4 as the maximum value of the abovementioned Likert scale, the interface scored a mean of 3.16 (SD 0.45; median of the means 3.2) for layout and visual design and a mean of 3.43 (SD 0.33; median of the means 3.51) for navigation and tasks (the data are presented in detail in Tables 1 and 2). Within the chosen criteria, these results can be acknowledged as confident positive evaluations of the proposed interface.

**Table 1:** Layout and visual design checklist and respective peer review test results.

Checklist item		Evaluation						
		T^a^1	T2	T3	T4	T5	Mean (SD)	Median
1	Displayed content density is appropriate for target users and their tasks	3	4	2	4	4	3.4 (0.89)	4
2	The layout helps to keep the focus of attention on what to do next	3	3	2	2	4	2.8 (0.84)	3
3	There is a clear “starting point” for each screen	3	3	3	3	4	3.2 (0.45)	3
4	The app is pleasant to look at	3	3	2	3	3	2.8 (0.45)	3
5	The app has a consistent and clearly recognizable appearance that will interest users	4	3	2	3	4	3.2 (0.84)	3
6	The different app screens share a consistent layout	3	4	3	3	4	3.4 (0.55)	3
7	Related information and functions are grouped and clearly recognizable	4	4	2	3	4	3.4 (0.89)	4
8	The screens respect a grid of horizontal and vertical alignments	4	4	3	3	4	3.6 (0.55)	4
9	There is a good balance between information density and white space	4	3	3	2	3	3.0 (0.71)	3
10	Colors work well together, and the use of complicated backgrounds is avoided	3	4	2	3	3	3.0 (0.71)	3
11	Colors are used to structure and group items	4	2	2	3	2	2.6 (0.89)	2
12	The use of contrasting elements (eg, bold text) is applied to emphasize important topics/ or categories	4	4	2	2	3	3.0 (1)	3
13	The screens are organized well and have no irrelevant information	4	4	4	3	4	3.8 (0.45)	4
14	Icons, pictograms, and graphics are recognizable and/or intuitive to understand (concrete and familiar)	3	3	2	4	4	3.2 (0.84)	3
15	The basic elements (screen titles, navigation items...) are easy to find	4	4	4	4	2	3.6 (0.89)	4
16	Attention-grabbing elements (eg, animations, bold colors, distinctive sizes) are used with caution and only when needed	2	3	3	2	2	2.4 (0.55)	2
17	Icons are visually and conceptually distinct but share a common harmony (clearly part of the same family)	3	4	2	1	4	2.8 (1.3)	3
18	Clickable contents (buttons) are clearly recognizable as such	3	3	3	3	4	3.2 (0.45)	3
19	The relationship between controls and their actions is obvious	3	4	4	3	4	3.6 (0.55)	4
20	Radio buttons and check boxes are used appropriately	1	4	3	3	3	2.8 (1.1)	3
21	Nonbutton items do not have button characteristics	2	4	4	3	3	3.2 (0.84)	3
22	Clickable items and content (buttons) include redundant labels or subtitles	1	3	1	1	3	1.8 (1.1)	1
23	The most important information is clearly displayed in the start zone (no need to scroll)	1	4	4	3	4	3.2 (1.3)	4
24	The app clearly shows when there is off-screen content that requires scrolling to view	1	2	4	3	3	2.6 (1.14)	3
25	Meaningful labels, functional background colors, and the use of margins and white space help the user identify distinct items	4	3	3	4	4	3.6 (0.55)	4
26	Typeface fonts are used consistently	4	3	3	3	4	3.4 (0.55)	3
27	Text fonts (typeface) are readable	2	4	3	4	3	3.2 (0.84)	3
28	Use of italic text is avoided	4	4	3	4	4	3.8 (0.45)	4
29	The app avoids extensive use of capitalized text	4	4	3	4	4	3.8 (0.45)	4
30	Textual content is neither too short nor too long	4	3	3	4	3	3.4 (0.55)	3

^a^T: Test.

**Table 2:** Navigation and tasks checklist and respective peer review test results.

Checklist item		Evaluation						
		T^a^1	T2	T3	T4	T5	Mean (SD)	Median
1	The app has no irrelevant, unnecessary, or distracting information	4	4	4	4	3	3.8 (0.45)	4
2	Excessive text, animations, or images have been avoided	4	3	4	4	4	3.8 (0.45)	4
3	The user does not need to use memory to scroll through the app	4	3	4	4	4	3.8 (0.45)	4
4	The main path is clear, avoiding distractions	4	3	4	4	3	3.6 (0.55)	4
5	The information is presented in a simple and natural way	4	4	2	3	3	3.2 (0.84)	3
6	The number of screens per task has been minimized	4	4	3	3	4	3.6 (0.55)	4
7	The app requires minimal scrolling and clicks	3	3	4	3	3	3.2 (0.45)	3
8	The app correctly anticipates the user's next intentions	3	3	4	3	2	3.0 (0.71)	3
9	The use of metaphors is understandable	3	3	4	3	3	3.2 (0.45)	3
10	If there is an image or icon alongside a button, it is relevant for the task	3	4	3	4	4	3.6 (0.55)	4
11	Commands and actions are presented as buttons or gestures, not as hyperlinks	4	3	4	3	4	3.6 (0.55)	4
12	A new user can use the most common functions without assistance	3	4	4	3	4	3.6 (0.55)	4
13	There is a convenient and obvious way to go through the different screens of the app	4	4	3	3	4	3.6 (0.55)	4
14	The most relevant information is easily accessible	2	4	3	3	4	3.2 (0.84)	3
15	Navigation is organized in the most logical way and oriented to the app’s tasks	4	4	4	3	2	3.4 (0.89)	4
16	The structure of the app is simple and without unnecessary levels	4	4	4	3	4	3.8 (0.45)	4
17	The main sections of the app are available from any screen and there are no dead ends	3	2	4	2	2	2.6 (0.89)	2
18	Navigation feedback is appropriate	3	3	4	3	3	3.2 (0.45)	3
19	The app has its own consistent graphic terminology and conventions between the different screens	3	4	2	3	3	3.0 (0.71)	3
20	Only navigation screens (such as the homepage) can be viewed without scrolling	3	4	2	4	4	3.4 (0.89)	4
21	The app allows the user to browse at their own pace	3	4	4	4	4	3.8 (0.45)	4

^a^T: Test.

Nonetheless, some parameters scored moderately high values: *22. Clickable items and content (buttons) include redundant labels/subtitles* for layout and visual design was the lowest scored item, with a mean evaluation score of 1.8 (median 1, SD 1.1). Other low-score parameters include *11. Colors are used to structure and group items* (mean 2.6, median 2, SD 0.89) and *16. Attention-grabbing elements (eg, animations, bold colors, different sizes) are used with caution and only when necessary* (mean 2.4, median 2, SD 0.55).

The lowest score for the navigation and tasks scope concerned the parameter *17. The main sections of the app are available from any screen and there are no dead ends* (mean 2.6, median 2, SD 0.89). The following lowest value already achieved an appreciably positive value of 3/3 for *8. The app correctly anticipates the user’s next intentions*.

Although the quantitative approach provides important hints on what to look for and an overall assessment, the open-ended commentaries enable better understanding of the reviewer’s concerns. In this section, layout and visual design received a mean of 1.2 comments per parameter (median 1); the abovementioned parameter 16 was the one that received the most feedback, with comments from 4 of the 5 peer reviewers. All comments for this parameter warned about different issues such as text sizes, contrasts, visual weight, and sparse use of colors. The parameter *11. Colors are used to structure and group items* had 3 comments, all of which highlighted the absence of a more variable color palette (eg, “Color variability is not something that goes into this app”). In addition, with 3 comments, the parameter *20. Radio buttons and check boxes are used appropriately* raised issues concerning the interaction limitations of the mockup.

The navigation and tasks section received approximately 0.714 comments per parameter (median 1). The most frequently commented parameter (3 comments) was *17. The main sections of the app are available from any screen and there are no dead ends*. The comments reflected on the one-way-path aspect of the app, the limitation of the mock-up in turning back one action, the lack of a submission confirmation page (“I would say that before presenting the likely diagnosis there should be an confirmation of intention to ‘submit’”) and the difficulty in locating the button to go back to the homepage and repeat the test (“I can’t find a way to go back to something that resembles the homepage”).

## Discussion

### Principal Findings

In this study, we present the main stages and outcomes of the current developments in the interface design for the eHealthResp mobile app as a means to produce guidelines for mobile interface development for other CDSS tools with similar characteristics. Guidelines such as these, though available, are still scarce and lack validation [60].

The literature review highlighted the possibility and need for mHealth solutions to enhance diagnosis [13,26-28,31,33,35,40,42,50,52,60]. It gave a glimpse of the large number of mobile apps currently available within the health topic [43-45] and also flagged the lack of cohesive evaluation standards among them [25,26,28,38,41,60]. However, it helped collect important guidelines to foster better CDSS and mHealth solutions [13,15,18,19,27,30,36,44,46-52,61] to positively impact the quality of care regarding diagnosis [47] and potentially support overburdened medical education programs, promoting better patient care [30] and better, quicker, and more confident clinical decision processes by physicians [35].

As one of the most prominent studies found in the literature, Mobile Airways Sentinel networK (MASK), part of the ARIA initiative, focuses on the design and implementation of tools and guidelines for tool development in the scope [13,15,16,18-20,61].

Within several references to the ARIA project, Courbis et al [19] described a cascade-like methodology for implementing clinical decision support from paper guidelines to the MASK mobile app. A similar approach was adopted in building the eHealthResp app, including collaborative ways of designing and evaluating the solution and transforming the validated algorithms into a user-friendly interface.

The eHealthResp mobile interface design also follows a very similar design methodology to that adopted in mPneumonia [46]. The study makes use of prevalidated algorithms transforming them from paper into a step-by-step, user-friendly assessment questionnaire for mobile interfaces. The team also focused on the feasibility and usability, and unlike our study, they managed to gather acceptability levels. Most of the problems raised in the mPneuomonia project were conveniently approached while designing the first version of the eHealthResp mobile interface and were not raised during the appraise by the peer reviewers.

In accordance with the literature review, the state of the art review reiterates the existence of many apps available within the health care spectrum [43-45]. Despite this, these apps are presented through poor classification and evaluation systems [28], making it difficult to search for trustworthy and easy-to-use apps for a specific issue such as CDSS for upper respiratory infections.

Alongside conclusions by Panesar et al [50], we believe that a well-designed and accepted smartphone app can increase awareness of the importance of antimicrobial stewardship and influence some prescribing behaviors. The right information in the right context can reduce uncertainty, particularly in the antibiotic prescription realm [32].

Within the process of designing the interface, the most important factor was the design team’s interdisciplinary dimension. We tried to convey the warnings from researchers such as Litvin [49] and Rawson [51] about the need to predict the tool inclusion in the clinical workplace and grant the perception of usefulness to assist in decision making. This was done by directly involving the clinical scope throughout the design study from an early stage. This involvement was granted by an interdisciplinary design team with experienced members in the clinical field and also by targeting the app toward the pharmacists and physicians’ participants of the seminar (introduced in the Background section of this paper).

Despite confirming the beneficial outcomes for patients, Terry [28] flags the ongoing issue of classifying and rating mobile apps for health and calls for the inclusion of physicians, patients, and caregivers in the evaluation process. In addition, while detecting strengths, weaknesses, opportunities, and threats of smartphone-supported diagnosis for the particular case of allergy diseases, Pereira [26] highlights the lack of validation for this type of tool (for diagnostic decisions) and calls for multidisciplinary studies, similar to the research in focus on this paper, to obtain high-quality and useful tools.

These conclusions go along with the outcomes expressed throughout the design process, during which insightful outcomes arose from several meetings and questionnaires regarding preferences and worries predicted by the team members closer to the end-user’s community. Although some authors call for automated evaluation tools [36], we retained the use of questionnaires and meetings with experts to evaluate the designed app as a pragmatic way of making quick assessments and fostering the interaction between the design and pre-evaluation processes.

In addition, and because the contexts of use and users were already clearly predefined and represented among the research team members, it was possible to address the issues with a close to *contextual design* approach without the need for deep or direct research within the broad and complex scope of the study and all the limitations that this approach could imply [25].

At the same time, fast, interactive, and high-fidelity prototyping was revealed to be a major key factor for the co-design approach. This allowed other nonexpert designers to understand the approach almost seamlessly without requiring any kind of abstraction or written descriptions. Problems arose only with specific limitations of the prototyping tools, such as the inability to conveniently represent horizontal scroll, specific content animations (eg, icons mutations, element dislocations), and different screen aspect ratios.

Other interface design insights were taken into consideration; for example, Martínez-Pérez et al [44] highlight the need to avoid the use of text-only interfaces, making use of the interactivity, images, and logical decision trees throughout algorithms in a step-by-step approach to input data and restrict the input need to the minimum, reducing the time required to complete the diagnosis.

Guidelines for Android interfaces [53] were used to convey consistency with the primary system (Android) in which the mobile app will run. After the app is fully developed, convenient adaptations will be made so that the interface can be used equally in the iOS ecosystem [62].

In the scope of web design, Lindgaard [54] hints at the importance of immediately perceived esthetics, beauty, and visual appeal to grant hedonic values and urge the user to trust and use the tool. However, in the realm of web design, we believe that these values can be adopted in any design project that relies on the visual sense to obtain the user’s trust.

In a study by Shneiderman, the “golden rules of interface design” [59] were addressed throughout research by Gong and Tarasewich [55] to re-adapt these rules into “guidelines for handheld mobile device interface design.” These guidelines comprise original, adapted, and new guidelines that enable frequent users to use shortcuts; offer informative feedback; design dialog to yield closure; support internal locus of control, consistency, reversal of actions, error prevention and simple error handling; reduce short-term memory load; design for multiple and dynamic contexts; design for small devices; design for limited and split attention; design for speed and recovery; design for top-down interaction; allow for personalization; and design for enjoyment.

Nielsen and Budiu [58] highlight the general characteristics of mobile human-computer interactions. Among other things, they underline the importance of a clear start-up screen and the consistency between app pages and branding.

The authors also bridged the design stage to the evaluation stage, often linking these stages in a circular manner that fosters redesigning and re-evaluation. Authors such as Kushniruk et al [48], Shneiderman et al [59], authors from the Interaction Design Foundation [56], Nayebi et al [57], Nielsen and Budiu [58], and Travis [11] stress the importance of good usability testing, qualitative research, and methodologies, including usability heuristics with well-tested design principles for inspection, walkthroughs, action research methods, and concepts such as affordance.

Despite all the evidence that mobile devices are valuable tools for clinical decision-making by both physicians and pharmacists, there is still a need for rigorous evaluation, validation, and best practices for development to ensure the end-quality and safety of the tool [33]. Although the literature was very positive regarding the use of mobile technologies, it also warned about user anxiety issues, limited access to technologies for some, and security concerns [37].

A peer review was necessary to understand the interface limitations in a broad scope. The adopted methods and tools were revealed to be useful and suitable to quickly assess the reviewers’ opinions about the interface issues. Overall, the 5 reviewers provided approximately 0.96 comments per parameter, reflecting considerable efficiency of the chosen parameters in raising potential problems. As the main research goal was to detect potential issues rather than to conduct an overall evaluation, the adaptation of the guidelines by Travis [11] enabled a quick and efficient evaluation setup. The shortening of the classic 5-point Likert scale to 4 points allowed a relatively short sample of peer reviewers to express their opinions in a more binary (positive or negative) way for each guideline. The downside of this new scale concerns the negative assessment of some bullet points that could otherwise be classified as neutral evaluation and the overlooking of some positive points. Despite this concern, the reduced number of reviewers and the need to interpret every comment made allowed us to carefully assess each bullet point.

**Figure 3:**
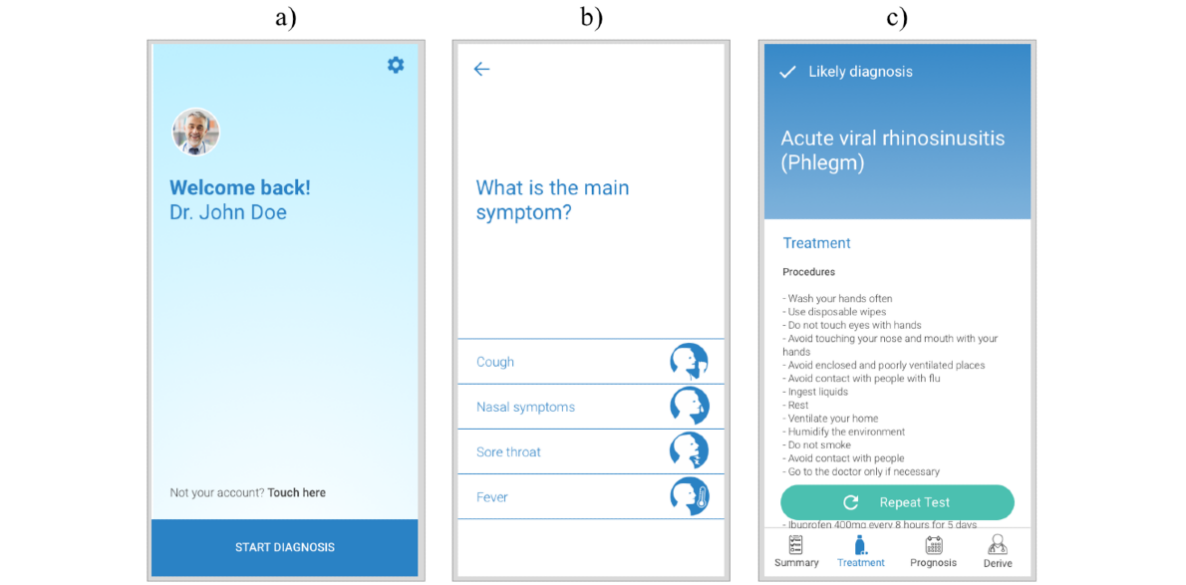
Light theme interface redesign examples after evaluation. (a) Homepage; (b) first question page; and (c) likely diagnosis page. Note: English versions made specifically for the purposes of this paper.

The results supported the creation of the beta version of the interface (Figure 3), addressing most of the detected problems. The version was further reassessed by 2 of the previous reviewers, validating the new design. In this second stage of validation, performed by 2 of the more critical reviewers, the interface was classified as clear and easily usable.

**Figure 4:**
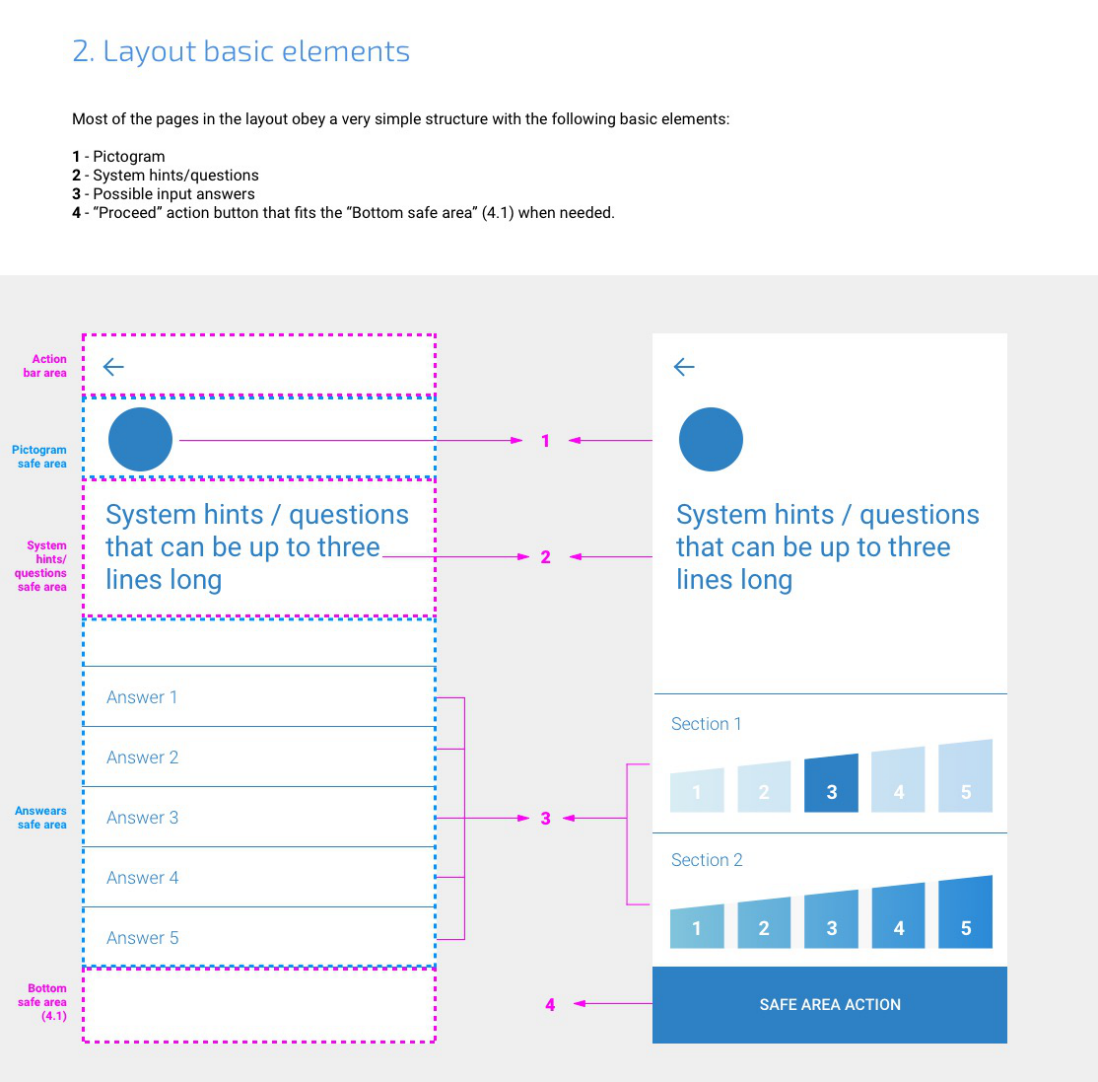
Example page of the design-development guidebook (Chapter: Layout basic elements).

Overall, the processes of co-design, evaluation, redesign, and re-evaluation produced valuable outcomes, addressing major problems of the initial design and proving the processes to be an efficient strategy to speed up the design process. As the main outcome, the study enabled us to create a guidebook for the development, with recommendations (Figure 4) explaining the rationale behind the design choices and the constructive rules for the interface. This guidebook allows developers to have a clear perception of the composition of the interface, summarizing the following chapters: (1) introduction, (2) layout basic elements, (3) page types, (4) specific layout behavior, (5) grid or relative distances, (6) themes, (7) color scheme, (8) typeface, (9) list of pictograms, (10) transitions between pages, and (11) animations.

### Limitations

The major limitation of the study is the relatively small design team. Although the small team size contributed to speeding up the decision processes, including only 2 HCI experts and 3 experts in pharmacology and pharmacoepidemiology could preclude the representation of the variety of possible contexts of use.

As already highlighted in the previous sections, limitations within the interactive mock-up also presented an issue to the nondesigner team members and reviewers’ assessment in independently interpreting the solution. Specific limitations of the prototyping tools in representing items such as horizontal scrolling, specific content animations (eg, icons mutations, element dislocations), and different screen aspect ratios, caused some confusion in interpreting the solution. In addition, the fact that some buttons being represented were not interactive and led to no alternative path caused some uncertainty regarding the true meaning of these elements among the reviewers and nondesigners.

These limitations could only be clarified in person and within the second evaluation stage with the reviewers.

### Future Work

Future work should include the implementation of the app and its confrontation with established assessment criteria such as mHealth evidence reporting and assessment [63].

After this step, usability tests with end-user input must be performed. Conveniently selected physicians and pharmacists in their workplaces will assess the usability of the mobile app using the System Usability Scale as a tool. Their feedback and adherence will better ascertain the effectiveness of all solutions [41].

After the design is revised and established, the research will undertake a pilot study to evaluate the effectiveness of the aforementioned tools, covering 20 primary care physicians, 20 community pharmacists, and 50 patients selected by key informants. This study will gather quantity and quality indicators as response variables within the context of antibiotic consumption to be statistically analyzed on an intention-to-treat basis. The tool should undertake a validation, in which the outcomes from the use of the mobile tool will be compared with those within the usual clinical decision setting (without the tool) together with a user survey regarding the user experience and usefulness of the app [23].

### Conclusions

Understanding the true impact of mHealth tools is still an uncertain task, as Forrest et al [39] concluded from their analysis of CDSS solutions. At the time of the study, it was still difficult to perceive the true impact of these tools on patient health outcomes. Although these solutions can significantly improve adherence to antibiotic prescription guidelines, providing easy access to these tools may not be sufficient to achieve higher levels of adherence [34].

As deepened in the literature and state of the art reviews, there is a need to develop recognizable standards in the development of mHealth solutions for CDSS in upper respiratory symptoms control. For this, the main factors influencing the success of these tools must be identified to complement the existing guidelines for mobile development [13,42,44,50-55]. More specifically, there is a need to define requirements relating to layout and content design for usability, acceptability, and usefulness of the app contents and features.

In an attempt to answer the question “How do we develop an interface for an app to support physicians, pharmacists, and patients to properly use previously validated algorithms for upper respiratory symptoms?” the research performed to date has contributed to clarifying some relevant aspects. The use of state-of-the-art tools for high-fidelity prototyping can be crucial to speed up the design process for a multidisciplinary team, not only because it can clearly represent the designers’ conventions to the rest of the team but also because it can help to easily and quickly integrate ongoing suggestions, allowing for a highly interactive co-design process that conveys the team’s concerns.

The developmental methodology enabled us to produce a set of guidelines or templates to produce an app that conveys the requirements of the app and aids the implementation stage.

## Abbreviations

ARIA: Allergic Rhinitis and its Impact on Asthma
CDSS: clinical decision support system
HCI: human-computer interaction
MASK: Mobile Airways Sentinel networK
mHealth: mobile health
